# The unidirectional phylogeny of *Homo sapiens* anchors the origin of modern humans in Eurasia

**DOI:** 10.1186/s41065-021-00197-7

**Published:** 2021-09-14

**Authors:** Úlfur Árnason

**Affiliations:** 1 Department of Clinical Sciences Lund, Lund University, Lund, Sweden; 2 Department of Neurosurgery, Skane University Hospital in Lund, Lund, Sweden

**Keywords:** Human evolution, Molecular phylogenetics, Out of Eurasia hypothesis, OOEH, Out of Africa hypothesis, OOAH, mtDNA, nuDNA, Y-DNA

## Abstract

**Background:**

The Out of Africa hypothesis, OOAH, was challenged recently in an extended mtDNA analysis, PPA (Progressive Phylogenetic Analysis), that identified the African human populations as paraphyletic, a finding that contradicted the common OOAH understanding that *Hss* had originated in Africa and invaded Eurasia from there. The results were consistent with the molecular Out of Eurasia hypothesis, OOEH, and Eurasian palaeontology, a subject that has been largely disregarded in the discussion of OOAH.

**Results:**

In the present study the mtDNA tree, a phylogeny based on maternal inheritance, was compared to the nuclear DNA tree of the paternally transmitted Y-chromosome haplotypes, Y-DNAs. The comparison showed full phylogenetic coherence between these two separate sets of data. The results were consistent with potentially four translocations of modern humans from Eurasia into Africa, the earliest taking place ≈ 250,000 years before present, YBP. The results were in accordance with the postulates behind OOEH at the same time as they lent no support to the OOAH.

**Conclusions:**

The conformity between the mtDNA and Y-DNA phylogenies of *Hss* is consistent with the understanding that Eurasia was the donor and not the receiver in human evolution. The evolutionary problems related to OOAH became similarly exposed by the mtDNA introgression that took place from *Hss* into Neanderthals ≈ 500,000 YBP, a circumstance that demonstrated the early coexistence of the two lineages in Eurasia.

## Background

A continuous unidirectional evolution from the root of the tree to the tip of each individual branch is an indisputable requirement for the validity of any phylogenetic tree. In a recent study [1] the direction of evolution in the tree of modern man, *Homo sapiens sapiens*, *Hss*, and man’s closest extinct relatives was determined in accordance with this condition. The results were inconsistent with the common OOAH understanding that *Hss* had originated in Africa and invaded Eurasia from there.

In their study [1] the authors introduced a new molecular approach, Progressive Phylogenetic Analysis (PPA) that allowed establishment of the direction of evolution in the *Hss* tree. The results showed that the African *Hss* populations constituted a paraphyletic assembly, a finding that compromised the foundation of the OOAH. In comparison the established PPA phylogeny was consistent with the OOEH and the Eurasian palaeontology of both *Hss* and *Hsn*(*Hsnn* +*Hsnd*), a topic that has been largely ignored by the adherents of OOAH.

Here the evolution of *Hss* was addressed in the light of the phylogenies of two separate sets of data, viz. the nuclear DNA, nuDNA, tree of the paternally transmitted Y-chromosome haplotypes, Y-DNAs [2–4], and the mtDNA tree, a non-nuclear tree with maternal inheritance. The findings supported conclusively the results presented in the initial Y-DNA studies [3, 4] and the more recent mtDNA findings that have challenged OOAH [1, 5, 6].

During the latter part of the 1980s, fragmentary sequence data from mtDNA became introduced in molecular phylogenetics with the aim of establishing the relationships within and among different species. In this era the group of Allan C. Wilson presented results that, according to the authors [7, 8], provided evidence that *Hss* had originated in Africa and subsequently migrated from there into Eurasia. The hypothesis soon became the norm in the discussions of *Hss* evolution, although the molecular basis for the understanding was questionable and the palaeontological support for it was lacking. Furthermore, examination of the same data in other molecular studies [9–11] did not favour the trees upon which the OOAH postulate was proposed.

The extensive studies of human Y chromosome haplotypes referred to above [3, 4] have yielded comprehensive phylogenetic results that are highly relevant to the discussion of OOAH. Contrary to the common OOAH understanding these studies identified a basal divergence between the non-African and African *Hss* populations, a finding that was incompatible with the OOAH position of a late *Hss* exodus out of Africa.

Here the topologies of the Y-DNA tree [2, 4] and the PPA phylogeny of the mtDNA tree of *Hss* [1] were compared. Thus, the comparison comprised two evolutionarily separate sets of data, viz. a paternal nuclear marker, Y-DNA, and a non-nuclear marker, mtDNA, based on maternal inheritance. The comparison yielded results that were mutually consistent with each other and with the OOEH while they lent no support to the out of Africa hypothesis.

The Y-DNA evolution of Neanderthals and Denisovans was addressed in a recent study [12] that related the Neanderthal mtDNA replacement to an ancient gene flow from an early lineage related to modern humans. The authors [12] referred to two studies [13, 14] in this connection, both acknowledging *Hss* origin in Africa and OOAH. One of these studies [13] presented a comprehensive OOAH account that rested upon *Hs* origin in Africa followed by an African divergence between *Hss* and *Hsn* (Neanderthals/Denisovans). This stage was followed by an out of Africa exodus of *Hsn* and a Eurasian divergence of *Hsn* into Neanderthals and Denisovans and a late out of Africa exodus of *Hss*. These circumstances have limited support in palaeontology and molecular findings related to *Hs*(*Hss* + *Hsn*) evolution.

## Results and discussion

### The out of Eurasia phylogeny

**Fig. 1:**
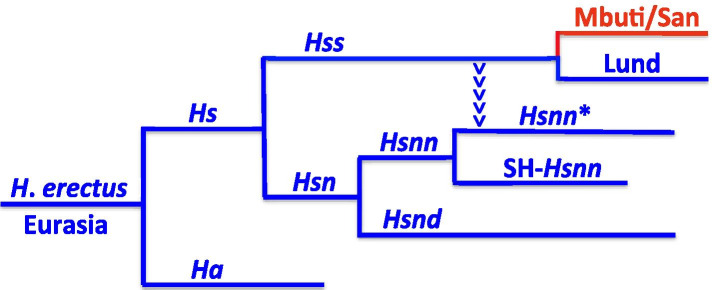
The nuDNA phylogeny leading to *Hss*, *Homo sapiens sapiens*. Blue: Eurasian lineages. Red: African lineages. *H. erectus* has been placed at the root of the tree in accordance with the artefact sequence related to the Eurasian existence of *He* 2,12 MYBP [15]. The divergence between *Hs*, *H. sapiens*, and *Ha*, *H. antecessor*, has been dated to ≈ 850,000 YBP [16, 17], that between *Hss* and *Hsn*, *H*. *s*. *neanderthalensis*, to ≈ 800,000 YBP and that between Lund and Mbuti/San to ≈ 250,000 YBP. *Hsn* divides into *Hsnn*, Neanderthals proper, and *Hsnd*, Denisova, with *Hsnn* dividing further into SH-*Hsnn* (*Hsnn* at Sima de los Huesos) and Hsnn*, a branch arising as the result of the mtDNA introgression that took place from *Hss* into Hsnn* ≈ 500,000 YBP [1]

Figure 1 outlines basal *Hs* relationships in accordance with recent advances in Eurasian palaeontology and phylogenetics [1]. The Eurasian-derived parts of the figure are marked blue and the African contribution red. *Homo erectus*, *He*, has been placed at the root of the tree consistent with *He* entering Eurasia from Africa > 2 million years ago [15] in agreement with the Eurasian palaeontology of the species. As apparent the evolution is continuous and unidirectional through the entire tree from the oldest to the most recent *Hss* divergence as represented by the African Mbuti/San and the Eurasian Lund.

 The earliest divergence in Fig. 1, that between *Ha*, *Homo antecessor*, and *Hs*, *Homo sapiens*, has been placed palaeontologically [16] and molecularly [17] at ≈ 850,000 YBP. As Fig. 1 shows, the *Hs* branch splits into a branch leading to extant humans, *Hss*, and another branch, *Hsn*, *H. sapiens neanderthalensis*, that gave rise to *Hsnn*, Neanderthals proper, and *Hsnd*, Denisovans. *Hsnn* diverged early into two branches, SH-*Hsnn* and Hsnn*, both palaeontologically and molecularly [18] identified. SH-*Hsnn* inhabited northern Spain, while Hsnn* reigned both in Europe and Asia, as established by extensive Eurasian fossil finds.

**Fig. 2:**
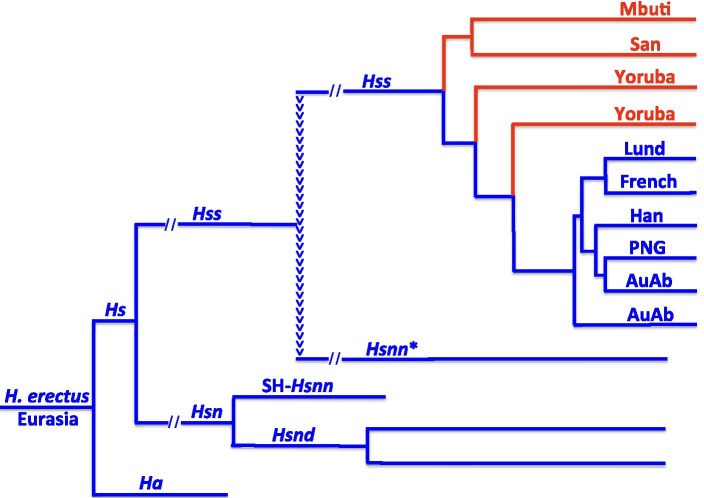
The mtDNA relationships of *Hs* demonstrating the paraphyly of the African *Hss* populations as resolved by PPA. Blue: non-African taxa; red: African taxa. Hsnn*: *Hsnn* other than SH-*Hsnn*. The arrowheads signify the mtDNA introgression that gave rise to Hsnn*. The limitation of *Hsnn* to Eurasia places the mtDNA introgression in this continent, reversing the direction of *Hss* evolution behind OOAH. The *Hss* part of the tree underlines the phylogenetic continuity among non-African populations and the paraphyly of the African populations including the two Yoruba [1]. AuAb: Australian aborigines; PNG: Papua New Guinean; Han: Chinese; Lund: The first described non-chimaeric human mtDNA molecule [19]; French: A European, as representing previous genomic findings [20]

The arrowheads that lead from *Hss* to Hsnn* in Fig. 1 mark the phylogeny resulting from the mtDNA introgression that took place from *Hss* into Hsnn* ≈ 500,000 YBP [1 As shown in Fig. 2 the introgression joins *Hss* and Hsnn* on a common mtDNA branch, therewith restricting the initial mtDNA branch of *Hsn* to SH-*Hsnn* and *Hsnd*. With the Neanderthals strictly limited to Eurasia it becomes apparent that the mtDNA introgression from *Hss* to Hsnn* could only take place in conjunction with the contemporary coexistence of both *Hss* and *Hsnn* in Eurasia [1, 6], a circumstance that invalidates OOAH since that hypothesis does not allow the existence of *Hss* in Eurasia at the time in question.

An extensive genomic study of extant humans that was presented a few years ago [20] identified a basal *Hss* divergence between a Eurasian (French) genome and the genome of the African Mbuti, but the significance of the finding for addressing *Hss* origin and evolution was not discussed. However, as maintained subsequently on phylogenetic grounds [6], this basal *Hss* divergence compromised OOAH since that hypothesis rested instead upon an *Hss* exodus out of Africa into Eurasia by a late arising African population and not on a population that constituted the earliest divergence among recent humans.

In the recent study by Árnason and Hallström [1] the direction of evolution in the *Hss* tree was established by applying a new approach, Progressive Phylogenetic Analysis, PPA, which demonstrated that the African populations constituted a paraphyletic grouping. The African paraphyly invalidated the postulate of an *Hss* origin in Africa and a late exodus out of that continent into Eurasia since the PPA identified instead a minimum of three separate waves of *Hss* migration from Eurasia into Africa the earliest being that of the ancestors of Mbuti/San. Thus, in contradiction to OOAH the PPA approach yielded results that were consistent with a continuous unidirectional Eurasian evolution on the *Hs* branch from the divergence between *H*. *antecessor* and *H*. *sapiens* to the apical tips of extant *Hss* as demonstrated in Figs. 1 and 2.

### The profiles of the Y-DNA and mtDNA phylogenies of *Hss*

**Fig. 3:**
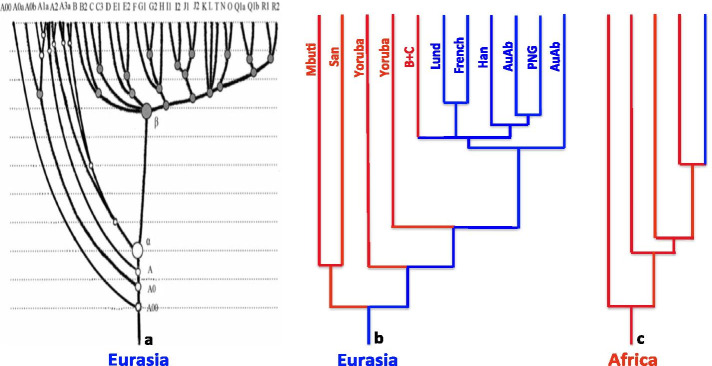
The Y-DNA and mtDNA phylogenies of *Hss* with the Y-DNA tree (**a**) [4] and the mtDNA tree (**b**) [1] representing the OOEH phylogeny, and tree (**c**) standing for the OOAH phylogeny of both mtDNA and Y-DNA. Trees (**a**) and (**b**) are consistent with a residing Eurasian *Hss* populations and a series of *Hss* exoduses from Eurasia into Africa. Position A00 in the Y-DNA tree and the corresponding position in the mtDNA tree mark the position at which the basal African and non-African lineages of extant *Hss* populations coalesce. Position β in tree (**a**) signifies the beginning of the Eurasian diversification of *Hss* dated to ≈ 125,000 to 120,000 YBP in the mtDNA tree [1]. The blue branch in (**c**) signifies a late *Hss* exodus out of Africa as assumed by the out of Africa hypothesis. As underlined in Figs. 1 and 2, the position of the root of the phylogeny in tree (**c**) is without connection to the Eurasian evolution of *Hss* and *Hsn*(*Hsnn* + *Hsnd*)

Figure 3a–c shows the phylogenetic relationships of recent *Hss* as resolved in analysis of two separate sets of data, the paternally transmitted Y-DNA and the maternally transmitted mtDNA. The Y-DNA tree of OOEH [3, 4] is shown to the left in the figure, the mtDNA tree of OOEH in the middle [1] and the commonly acknowledged Y-DNA tree of OOAH (e.g. [2]) to the right. Although the three trees are superficially similar they are fundamentally different in that trees 3a and 3b are consistent with the out of Eurasia hypothesis whereas tree 3c is that of OOAH.

The extensive Y-DNA study [4] behind Fig. 3a showed a minimum of five waves of *Hss* migration from Eurasia into Africa while the more limited mtDNA sampling behind 3b [1] identified a minimum of three migrations with each of these coinciding with the Y-DNA results, consistent with the presence of both males (Y-DNA) and females (mtDNA) in each population migrating into Africa. As apparent a divergence between Mbuti and San prior to their migration into Africa [5] would raise the number of waves into Africa by one, as would also any migration connected to the B + C branch [4] that has been tentatively indicated on the mtDNA tree.

With respect to the phylogeny in Fig. 3c it should be noted that the OOAH tree and the out of Africa hypothesis, in addition to their earlier rebuttal [3, 4], became rejected by the recent PPA findings [1] which demonstrated the paraphyly of the African populations. In contradiction to OOAH these molecular results were all in accordance with the out of Eurasia hypothesis and the Eurasian palaeontology of both *Hss* and *Hsn*(*Hsnn* + *Hsnd*), a topic that has been largely ignored by the adherents of OOAH.

The Y-DNA tree in Fig. 3a and the mtDNA tree in 3b are both consistent with a unidirectional *Hss* evolution from the Eurasian root of the *Hs* tree to the tip of each individual *Hss* branch. The Y-DNA tree is based upon the largest sample that has been used to delineate the Y-DNA relationships of extant humans. The analysis [4] identified a Eurasian Y-DNA coalescence, A00, that was followed by three separate *Hss* exoduses, A0, A and α, from Eurasia into Africa with A0 signifying the earliest and α the most recent of these early exoduses. The Eurasian Y-DNA phylogeny shows a continuous Eurasian span from position α to the extensive Eurasian diversification beginning at position β in the Y-DNA tree.

The mtDNA tree in Fig. 3b mirrors the Y-DNA phylogeny in accordance with a shared identity, male and female, within each pair of the *Hss* exoduses from Eurasia into Africa. Similarly the barren Eurasian branch between the last exodus into Africa, and position β, the initiation of the Eurasian diversification of *Hss* is common to both phylogenies although the span is longer in the Y-DNA phylogeny than in the mtDNA tree, a distinction that might be related to different modes of calculation.

The phylogenetic position at position β in the Y-DNA tree was discussed in the recent mtDNA study [1] in the context of climatic cycles, the most severe of these ending ≈ 125,000 YBP. This climatic circumstance coincides with the restricted molecular variation at the corresponding position in the Eurasian phylogeny prior to the striking population expansion occurring later in the two separate sets of molecular data behind the Y-DNA and mtDNA phylogenies.

## Conclusions

The Y-DNA and mtDNA phylogenies discussed here are consistent with a unidirectional *Hs* evolution from the Eurasian root of the *Hs* tree to each individual branch connected to *Hss* evolution, including the populations that migrated from Eurasia into Africa. The findings compromise the large number of results that have been interpreted in accordance with the Out of Africa hypothesis and the a priori assumption that the African *Hss* populations constituted a monophyletic assembly from which Eurasia became colonized. A crucial phylogenetic circumstance related to the rebuttal of OOAH has been the identification of the separate *Hss* exoduses into Africa with each of these recorded by both the female inherited mtDNA and the male inherited Y-DNA.
